# Splicing regulatory factors in breast cancer hallmarks and disease progression

**DOI:** 10.18632/oncotarget.27215

**Published:** 2019-10-15

**Authors:** Esmee Koedoot, Liesanne Wolters, Bob van de Water, Sylvia E. Le Dévédec

**Affiliations:** ^1^ Division of Drug Discovery and Safety, LACDR, Leiden University, Leiden, The Netherlands

**Keywords:** hallmarks of cancer, breast cancer, alternative splicing, splice factors, RNA sequencing

## Abstract

By regulating transcript isoform expression levels, alternative splicing provides an additional layer of protein control. Recent studies show evidence that cancer cells use different splicing events to fulfill their requirements in order to develop, progress and metastasize. However, there has been less attention for the role of the complex catalyzing the complicated multistep splicing reaction: the spliceosome. The spliceosome consists of multiple sub-complexes in total comprising 244 proteins or splice factors and 5 associated RNA molecules. Here we discuss the role of splice factors in the oncogenic processes tumors cells need to fulfill their oncogenic properties (the so-called the hallmarks of cancer). Despite the fact that splice factors have been investigated only recently, they seem to play a prominent role in already five hallmarks of cancer: angiogenesis, resisting cell death, sustaining proliferation, deregulating cellular energetics and invasion and metastasis formation by affecting major signaling pathways such as epithelial-to-mesenchymal transition, the Warburg effect, DNA damage response and hormone receptor dependent proliferation. Moreover, we could relate expression of representative genes of four other hallmarks (enabling replicative mortality, genomic instability, avoiding immune destruction and evading growth suppression) to splice factor levels in human breast cancer tumors, suggesting that also these hallmarks could be regulated by splice factors. Since many splice factors are involved in multiple hallmarks of cancer, inhibiting splice factors might provide a new layer of oncogenic control and a powerful method to combat breast cancer progression.

## INTRODUCTION

During gene transcription, a pre-mature messenger RNA (pre-mRNA) molecule is generated that requires further processing to a mature in a mRNA molecule that will be translated into a protein. In this maturation step, the introns are usually removed and the exons are ligated. This process, called splicing, is one of the post-transcriptional processes essential for RNA translation into functional proteins and requires the activity of the splice factors. Besides simple intron removal and exon coupling, the activity of those splice factors enable that multiple protein isoforms can be translated out of one pre-mRNA transcript by selective incorporation of pre-mRNA parts in the mature mRNA transcript [1–4]. This is called alternative splicing (AS) and provides an essential layer of post-transcriptional regulation that only recently received much attention from the research community. In particular, it is becoming clear that tumor cells benefit greatly from this flexible regulatory process since many specific isoforms have been identified as promoting and supporting neoplastic transformation, tumor growth and progression. Many reports have linked AS to up-regulation of proto-oncogenes, deregulated cell division, increased survival, altered metabolism, onset of angiogenesis, increased invasion and metastasis in different cancer types including breast cancer [5–8].

Splicing is a complex multistep process catalyzed by the spliceosome, a large, dynamic, multicomponent complex consisting of five small nuclear ribonucleoproteins (snRNPs) U1, U2, U4, U5 and U6 and many associated proteins. In the human spliceosome, the 141 core factors are highly abundant and/or are specifically associated with the U1, U2, U5, U4/U6 snRNPs, or the U4/U6. U5 tri-snRNP [9, 10]. The auxiliary splice factors that are not part of the core spliceosome regulate AS and are less abundant when co-purified with the core spliceosome members [9, 10]. Splice factors are highly diverse considering both function and structure. For example, hnRNPs are characterized by a RNA Recognition Motif (RRM) domain that accommodates site-specific binding to the target RNA typically resulting in splicing inhibition by suppressing assembly of the spliceosome [11] or attraction of snRNPs [12, 13]. SR splice factors contain a domain consisting of arginine/serine repeats (RS domain) and at least one RRM domain [14–16] and facilitate recruitment of the snRNPs to the splice sites [2]. Additionally, their activity is regulated through phosphorylation by SR protein kinases (SRPKs). Currently almost 250 splice factors distributed over different classes have been identified, all playing a specific role at a specific stage of the splicing process [9, 17, 18]).

Breast cancer is the most frequent type of cancer in women with an estimation of 268,670 new cases and 41,400 deaths in the United States in 2018 [19]. In order to develop and progress, (breast) cancer cells move through various steps to fulfill their requirements for certain oncogenic properties. These processes – the so-called ‘hallmarks of cancer’ – have been summarized by Hanahan and Weinberg in 2000 and 2011 [20, 21] and currently include ten processes essential for tumor development and progression. In this review, we will discuss the spliceosomal changes across the different hallmarks of breast cancer. Since many of the already known spliceosome target genes have already been reviewed extensively elsewhere [5–8], we will focus on the role of splice factors as potential oncogenes or tumor suppressors in breast cancer. We will highlight newly identified splice factors of which abnormal regulation is linked to the different hallmarks of breast cancer [21]. For the hallmarks that have not yet been linked to splice factors expression, we identified factors strongly related to hallmark-specific oncogenic processes using publicly available RNA sequencing data. Finally, we discuss the clinical relevance of using splice factors as biomarkers and potential targets in breast cancer therapy.

## SPLICE FACTOR DYSREGULATION IN BREAST CANCER

Cancer-specific splicing events are established via different routes: 1) changes in expression levels, activity and localization of splice factors and/or 2) mutations in functional domains of splicing related proteins and/or mutations in regulatory sequences, such as enhancer/silencer sequences and branch points [22, 23]. Both processes can result in differential splice factor activity leading to differential splice site usage or increased or suppressed intron or exon inclusion. Those deregulatory events in splicing have been shown to play a prominent role in breast cancer.

### Altered expression, activity and localization of splice factors

#### Altered expression

In two independent studies, the comparison of the transcriptome of human breast tumors versus matched healthy tissue revealed that 10%–50% of the protein-coding genes have altered transcript variant expression levels [24, 25]. These patient data are in line with recent *in vitro* findings that show a significant switch in splicing pattern during epithelial-to-mesenchymal transition (EMT) accompanied with a specific EMT splicing signature [26]. Interestingly, this shift in splicing pattern was correlated to the expression levels of specific splice factors; all three studies revealed splice factor RBFOX2 as one of the most differentially expressed between the epithelial and mesenchymal cell state [24–26]. Moreover, expression levels of MBNL1, QKI, PTBP1, ELAV1, HNRNPC, KHDRBS1, SRSF2 and TIAR were also linked to the mesenchymal state [24]. By applying a splicing motif analysis in EMT regulated alternative transcripts, Shapiro et al. concluded that the MBNL, CELF, hnRNP, or ESRP splice factors were most likely involved in the EMT splicing patterns [26]. Furthermore, depletion of the mesenchymal splice factor RBFOX2 or overexpression of the epithelial factor ESRP in mesenchymal cells induced a more epithelial morphology and reduced cell motility [26]. Altogether, these data clearly suggest that splice factors can be in control of EMT and breast cancer progression.

#### Post-translational modifications and chromatin structure

Next to changes in expression levels, activity of certain splice factors can also be regulated by post-translational modifications (PTMs) such as acetylation, phosphorylation and ubiquitination. Strong interactions between ubiquitination and the spliceosome have been demonstrated and SR proteins are widely known to regulate the activation of other splicing factors by phosphorylation [27–29]. For example, acetylation and ubiquitination of the splicing factor SRSF5 has been shown to control tumor growth [30]. The phosphorylation status of SRSF1 and SRSF7 controls their function as only non-phosphorylated SR proteins were shown to facilitate the recruitment of mRNA to nuclear export receptors [31–34].

Moreover, the intracellular distribution can be crucial for downstream signaling events. Although most splice factors reside in the nucleus, cytoplasmic splicing has been recently shown to take place in mammalian cells implying that splice factors might have differential activities depending on their intracellular location [35]. Splice factor dynamics is also highly dependent on the chromatin structure that is often disturbed in cancer cells [36]. Non-coding RNAs (ncRNAs) and in particular long ncRNAs (lncRNAs) can alter the chromatin environment preventing the recruitment of a repressive chromatin-splicing adapter complex and consequently regulate AS of the FGFR2 [37]. Moreover, histone hyper acetylation has been shown to affect the distribution of several splicing factors such as SRSF1, SRSF2 SRSF3 and U2AF65, causing accumulation in the nuclear speckles [38] and decreased spliceosomal assembly at 3′ splice sites, while calcium-mediated histone hyperacetylation regulates AS of genes important in heart development [39]. Finally, splicing can be regulated by miRNAs within the supraspliceosome that can target different RNAs via alternative base pairing, thereby regulating gene expression and quality control of AS [40].

### Mutations in splice factors or regulatory sites

Next to altered splice factor expression levels and activity, abnormal splicing can be caused by mutations in the genes that encode these factors. Few studies and our own analysis (Supplementary Table 1) demonstrate that the splice factors U2AF1, SRSF2 and SF3B1 are often mutated in myelodysplastic syndromes [41], but also in solid cancers amongst which breast cancer [42]. These mutations mainly caused haematopoiesis due to impaired 3′-splice site recognition followed by RNA splicing deficiencies [41]. U2AF1 mutations specifically affected AS of genes in various pathways pivotal for cancer development, such as apoptosis via CASP8, DNA damage response via ATR and FANCA and DNA methylation via DNMT3B [43].

For the luminal breast cancer subtype, mutations in SF3B1 were found to be possible driver mutations [44–46]. These mutations result in a change-of-function and have been associated with hundreds of atypical splice sites at the 3′ end of the intron, thereby inducing AS of SF3B1 downstream target genes [47, 48]. Accordingly, our splice factor mutation analysis of breast cancer tumors from the COSMIC database revealed frequently mutated spliceosome genes amongst which SF3B1 (Supplementary Table 1). Interestingly, 10 splicing factors were classified as driver genes of which mutations are selected during tumor development by the Intogen database (Supplementary Table 1), 5.4% of all driver genes in breast cancer were regulating splicing, suggesting a major role for these proteins in breast cancer oncogenesis.

Next to mutations that could affect the functionality of splice factors, mutations in 5′- or 3′ splice site or branch point can disrupt or create splice sites [49] and thereby cause AS [50, 51]. Furthermore, specificity of AS is controlled by cis-regulatory elements that regulate the recruitment of trans-acting splicing factors to the splice site; exonic splicing enhancers (ESEs), exonic splicing silencers (ESSs), intronic splicing enhancers (ISEs) and intronic splicing silencers (ISSs) [2]. Mutations can modulate activity of these elements thereby affecting AS. For instance, a point mutation in exon 18 of important tumor-suppressor gene BRCA1 disrupts an ESE resulting in exon skipping [52], while mutations in the ESEs and branchpoint recognized by SRSF2 dysregulates spliceosome assembly and result in AS in myelodysplasia [53].

## ROLE OF SPLICE FACTORS IN THE HALLMARKS OF BREAST CANCER

**Figure 1:**
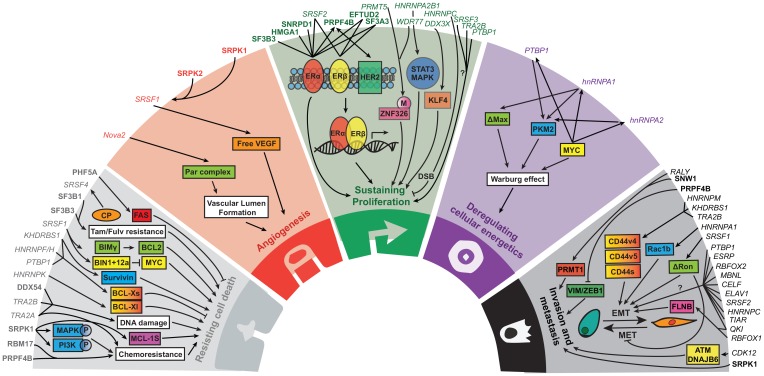
The role of splice factors and their associated pathways in the five hallmarks of cancer. Core splicing factors are listed in bold. Non-core splicing factors are listed in italic. Adapted from Hanahan and Weinberg, 2011 [21].

**Figure 2:**
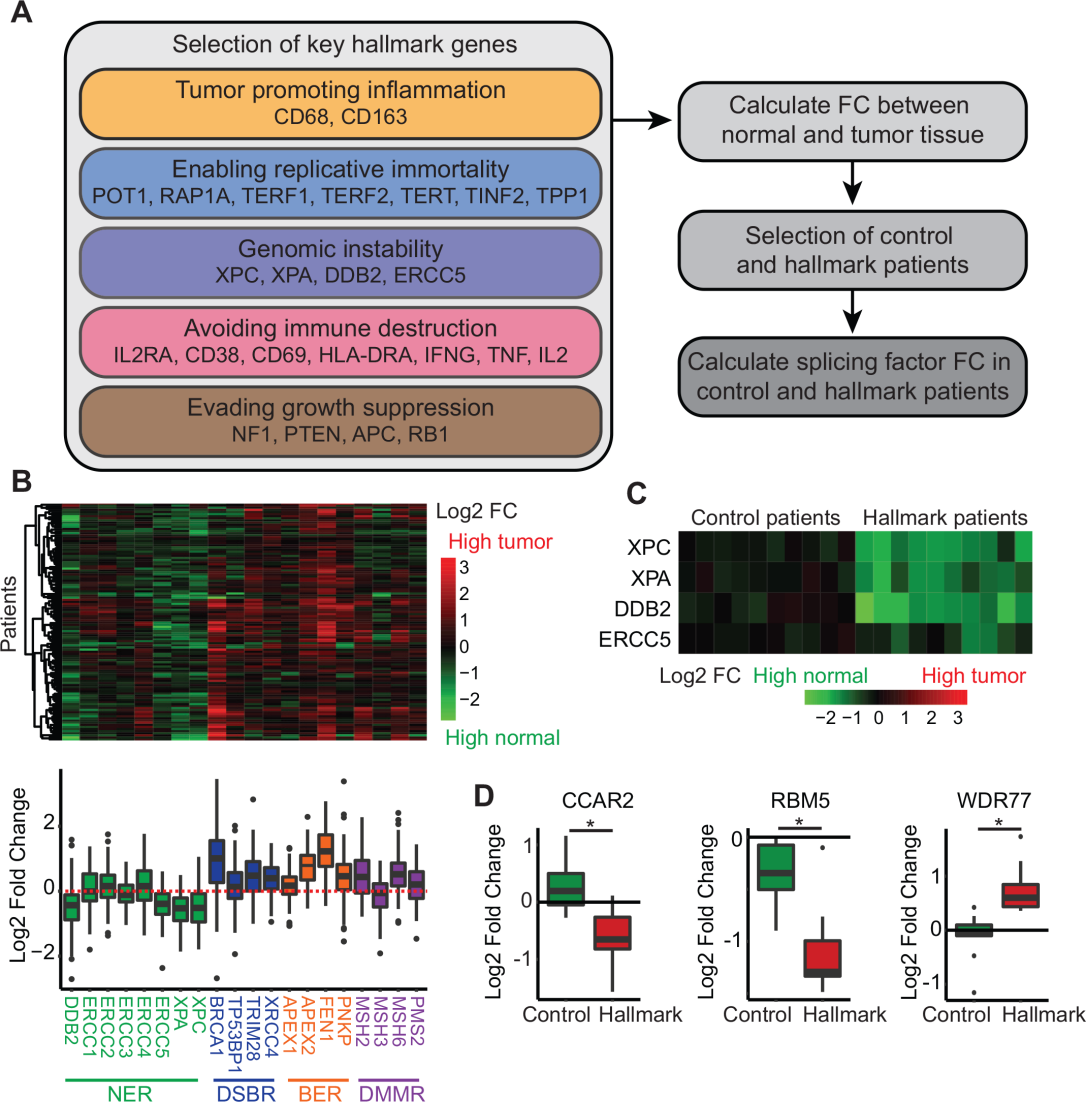
Relation of splice factor expression levels to other hallmarks of cancer. (**A**) Steps used to link splice factor RNA expression levels to the other hallmarks of cancer. (**B**) Heatmap displaying the log2 fold change of genome instability markers comparing primary tumor tissue to normal tissue. NER = nucleotide excision repair, DSBR = double strand break repair, BER = base excision repair, DMMR = DNA mismatch repair. (**C**) Heatmap of log2 fold change of genome instability markers in ten control and hallmark patients comparing normal to primary tumor tissue. (**D**) Log2 fold change of splice factors in control and hallmark patients comparing normal to primary tumor tissue.

In order to develop and progress, (breast) cancer cells move through various oncogenic processes. These hallmarks of cancer were summarized by Hanahan and Weinberg in 2000 and 2011 and now contain ten processes essential for tumor development and progression [20, 21]. Although the splice factor research in relation to breast cancer emerged only recently, there are already five hallmarks of cancer known to be affected by splice factors: sustaining proliferation, activation of invasion and metastasis, resisting cell death, deregulating cellular energetics and angiogenesis. Here we will discuss splice factors and up –and downstream pathways important in these five hallmarks (Figure 1, Supplementary Table 2). Moreover, we could relate expression of representative genes of four other hallmarks to splice factor levels in human breast cancer tumors (Figure 2, Supplementary Table 3), suggesting that also these hallmarks might be modulated by splice factors. However, the causal relationship between splice factor levels and these hallmarks of cancer remains to be elucidated.

### Sustaining proliferation

#### Hormone-receptor dependent pathways

Sustained proliferation is probably the most critical and studied cancer hallmark. Normal tissues precisely control cell number through many signaling pathways, amongst which the well-known MAPK and PI3K cascades [20, 54, 55]. In transformed cells, these pathways are upregulated resulting in uncontrolled growth. In breast cancer this mainly occurs via mutations resulting in overexpression of hormone receptors including the estrogen receptor (ER), androgen receptor (AR), epidermal growth factor receptor (EGFR), human epidermal growth factor receptor 2 (HER2) and their corresponding ligands [20, 56, 57].

The ER consists of two subtypes: ERα and ERβ. Upon ligand binding ERα and/or ERβ will form homo- or heterodimers, leading to nuclear translocation and possibly transcriptional activation [58]. ERα stimulates cell proliferation and survival by regulating the transcription of hundreds of genes [59, 60]. The role of ERβ in cancer has yet to be defined as several studies report contradictory correlations between transcript level and patient prognosis [61–63]. Emerging evidence suggests a role for splice factors in ER cancer signaling either by direct or indirect interactions with the receptors. For instance, RNA levels of SF3B3 correlate significantly with the overall survival of patients bearing ERα-positive tumors [64], PRPF4B protein expression levels are directly regulated by ERα activation [65] and HMGA1 is directly involved in ERα splicing [66]. Nassa et al. discovered that the interactome of both ERα and ERβ contains multiple splice factors of which some are common for both receptor subtypes (e. g. EFTUD2, SRSF2), while others only interact with a single receptor subtype, such as SNRPD1 for ERα and SF3A1 for ERβ [67]. Introduction of ERβ expression diminished 72% of ERα induced splicing events, but also introduced distinct splicing events in 28 genes that were functionally involved in many cellular processes including DNA replication and repair, DNA transcription, cell cycle and apoptosis [68]. Whether these changes are counteracting the proliferative effects of ERα transcriptional activity is yet unknown.

In addition to being regulated by ER signaling, PRPF4B levels are also controlled by HER2 signaling. Knockdown of HER2 results in downregulation of PRPF4B, whereas HER2 upregulation increases the levels of this splice factor in breast cancer cell lines [69]. Moreover, HER2 itself can be alternatively spliced resulting in different variants exhibiting either pro- or anti-tumorigenic functions. Skipping exon 16 results in the variant HER2D16, that is linked to increased resistance to HER2-targeting therapy and associated with cancer cell dissemination [70, 71]. Inclusion of intron 8 results in a truncated version of HER2 named Herstatin. Herstatin binds to the extracellular domain of HER2, preventing transfer to the cell membrane and receptor dimerization and phosphorylation, thereby exhibiting anti-tumor activities [72–74]. Although HER2 and ER expression patterns are inversely correlated because of negative feedback loops, they are interconnected via downstream pathways, such as the MAPK pathway. This crosstalk between ER and HER2 seems to be important in endocrine resistance [75] and therefore treatment might be improved by identifying more pathways or proteins regulated by both ER and HER2. Since splice factors like PRPF4B fit this double activation pattern, it might be worth investigating the role of PRPF4B in hormone receptor growth resistance conditions.

#### Hormone receptor independent pathways

Next to involvement in known proliferation pathways, splice factors are also implicated in tumor growth via partly unknown mechanisms that might be independent of these hormone cascades. Examples are PTBP1 which inhibits cell growth in breast cancer cell lines [76], Tra2β, a target of transcription factor and oncogene ETS-1 that is upregulated in breast cancer and is associated with cancer cell survival [77], SRSF3 that upon inhibition decreased breast cancer cell proliferation [78], the loss of PRMT5 or WDR77 resulting in AS and loss of proliferative genes [79] and HNRNPC which is highly expressed in breast tumors and of which knockdown results in double strand breaks and reduced proliferation [80]. Furthermore, DDX3× modulates the cell cycle by affecting splicing and expression of the cell cycle repressor KLF4, resulting in G1 arrest [81], while hnRNPA2B1 knockdown affects MAPK and STAT3 signaling resulting in prolonged S-phase [82]. Altogether, we can conclude that splice factors play an important role in breast cancer sustained proliferation by either directly or indirectly activating hormone receptors and other growth associated pathways.

Furthermore, in line with the existing concept of breast cancer cells being addicted to oncogenes such as MYC for their proliferative and survival capacity, few recent studies have reported the dependence of breast cancer on spliceosomal components [83, 84]. In general, the overall increase in gene expression in cancer cell implies their aggravated dependence on splicing factors which open up a new strategic window for targeting BC. Indeed, several splice factors including BUD31, SF3B1 and SRSF1 [85] are known to be gene targets of the oncoprotein MYC and inhibition or knockdown of those in MYC hyperactivated breast cancer cells impairs tumorigenesis [86].

### Activation of invasion and metastasis formation

Because the human breasts are non-vital organs, primary tumors in breast tissue can be surgically removed without major consequences. Ultimately, it is the formation of metastatic lesions in secondary organs that causes breast cancer mortality. Metastasis formation is often described as a multi-step process, also called the invasion-metastasis cascade [87, 88]. Primary breast cancer cells start to locally invade into the surrounding tissue and often those cells undergo a phenotypic switch characterized by epithelial-to-mesenchymal transition (EMT). In this process, epithelial cells bearing strong adhesion structures can switch to a migratory mesenchymal phenotype with loss of cell polarity and cell-cell contacts [89]. Those features acquired by mesenchymal cells allow not only infiltration into adjacent tissue, but also escape into the blood or lymphatic vessels.

Many splice factors have been linked to this EMT process. A genome-wide screen for EMT inducers identified many RNA-binding proteins, with splice factors QKI and RBFOX1 as main candidates. These factors regulated splicing of the actin binding protein FLNB, followed by release of FOXC1 leading to an intermediate mesenchymal cell state [90]. Moreover, the ratio of splice factors ESRP1/RBFOX2 was decreased during EMT and related to cancer progression and metastatic potential [91]. Next, the splice factor ESPR regulates fibroblast growth factor receptor 2 (FGFR2) splicing and thereby affects ligand binding, favoring FGFR2-IIIb which is specific to epithelial cells [92]. Genome-wide analysis of the ESRP splicing network uncovered hundreds of alternatively spliced genes that are involved in EMT related processes such as cellular adhesion and migration including ITGA6 and RALGPS2 [92].

Another important class of splice factors involved in metastasis formation are the heterogeneous nuclear ribonucleoproteins (hnRNPs), which can control splice site selection by either directly antagonizing the recognition of splice sites or interfere with proteins bound to enhancers [93]. For instance, cytoplasmic localization of hnRNPA1 is associated with metastatic relapse and activates RON translation that is known to affect cell migration and dissemination [94]. Conversely, nuclear hnRNPA1 acts as a tumor suppressor and inhibits exon 3b inclusion of the small GTPase Rac1, thereby repression formation of the Rac1b isoform [95]. Rac1b has a constitutively activated GTPase domain [96, 97] and is overexpressed in breast cancer [95, 98]. Matrix metalloproteinase-3 treatment interferes with hnRNPA1-Rac1 interactions, resulting in increased Rac1b levels and EMT [95, 99]. Moreover, breast cancer patients express high hnRNPA1 and low Rac1b levels in normal breast tissue, but low hnRNPA1 and high Rac1b levels in cancer tissue, suggesting that splicing of the Rho GTPase is also *in vivo* regulated by hnRNPA1 [95].

hnRNPM competes with the pro-epithelial splice factor ESRP1 for guanine-uridine rich motifs to regulate splicing of exons in genes involved in EMT-related pathways [100]. Furthermore, hnRNPM controls EMT by modulating CD44 isoform expression, which in turn increases TGFβ signaling. Elimination of hnRNPM prevents TGFβ induced breast cancer metastasis in mice by decreasing the mesenchymal-related standard CD44 isoform. hnRNPM mRNA levels were shown to correlate with aggressive breast cancer subtypes (basal and ER negative) and increased CD44 standard levels in breast cancer patients [101, 102]. Interestingly, the adhesion molecule CD44 that regulates the aggressive phenotype of breast cancer cells seem to be a common target of AS. KHDRBS1 is a factor involved in a dynamic protein complex variable in size and sensitive to EGF stimulation. EGF activation favors the smaller KHDRBS1 complex, which induces CD44 exon v5 inclusion resulting in enhanced cell migration [103]. Furthermore, SR splice factor TRA2β is overexpressed in invasive breast cancer and induces exon v4 and v5 inclusion [104], suggesting that besides the standard CD44 isoform also the v4 and v5 isoforms are related to increased invasion and metastasis formation.

The third hnRNP, PTBP1 is upregulated in progressively transformed human mammary epithelial cells (HMECs). Knockdown of PTBP1 impairs tumor cell growth, colony formation, *in vitro* invasiveness of breast cancer cell lines and transformation state of HMECs [76].

Next to the hnRNPs, the splice factor SRSF protein kinase 1 (SRPK1) was shown to be highly expressed in more aggressive basal breast cancer, correlating to less metastasis-free survival and specifically increased number of lung and brain metastases in patients. Stable knockdown of this kinase reduced metastasis to distant organs in a mouse model and inhibited focal adhesion reorganization, which were surprisingly not correlated to a downstream decrease in serine/arginine-rich (SR) splice factor phosphorylation [105]. However, an important role for these SR splice factors – in particular serine and arginine splice factor 1 (SRSF1) – cannot be excluded. SRSF1 is amplified and upregulated in breast cancer and transforms immortal cells when overexpressed [106, 107]. This transformation is mediated by SRSF1 collaboration with transcription factor MYC thereby amplifying eIF4E activation. This potential mechanism is further supported by patient data that reveal a significant co-expression of MYC and SRSF1 in human breast tumors [107]. Furthermore, SRSF1 mutants prevent tumorigenesis and soft agar colony formation by inhibiting activation of the B-Raf-MEK-ERK pathway [106]. Finally, SRSF1 activates EMT and cell migration by induction of DRon, a constitutively active isoform of the Ron tyrosine kinase receptor that is causally connected to EMT [108]. Interestingly, hnRNPA1 has been shown to antagonize SRSF1-mediated EMT activation: through the inhibition of DRon production, hnRNPA1 activates the MET program at distant sites, thereby enhancing metastasis formation [108].

Next to its oncogenic roles in sustaining proliferation, PRPF4B demonstrated an anti-oncogenic role in relation to EMT: loss of PRPF4B resulted in reduced EGFR degradation, increased expression of mesenchymal markers vimentin and ZEB1, detachment from the extracellular matrix and anoikis resistance [109]. Besides EMT, some splice factors have also been linked to the metastatic cascade in general, and their role in a specific step of the metastatic cascade remains to be elucidated. For example, RALY and SNW1 stimulate exon 2 inclusion in PRMT1, promoting breast cancer invasiveness [110] and CDK12 promote alternative last exon splicing of DNA damage genes ATM and DNAJB6 thereby increasing migration and invasiveness of breast cancer cells [111].

### Resisting cell death

#### Resisting physiological stresses

During tumorigenesis or anticancer therapy, cancer cells are exposed to numerous physiological stresses. In normal cells, these cellular stresses will cause apoptosis. However, cancer cells adapt in such environments and rewire their apoptotic program to survive. The SR related splice factor SRSF1 appears to play an important role in this process by promoting AS of crucial regulators of apoptosis BIM γ1 and γ2. Both isoforms lack the BH3 domain necessary to bind the anti-apoptotic Bcl-2 family members. Moreover, SRSF1 stimulates AS of a BIN1 isoform that is not able to bind MYC anymore, thereby losing its tumor suppressor activity leading to decreased levels of apoptosis [107]. This is in agreement with the observed upregulation of SRPK1 that contributes to the cytoplasmic accumulation of RNA-binding motif protein 4 (RBM4). This leads to the production of anti-apoptotic isoforms IR-A and MCL-1L and decreased sensitivity to apoptotic signals in breast cancer cells [112]. Furthermore, depletion of splice factor PHF5A increased apoptotic signaling by promoting expression of short truncated FAS-activated serine/threonine kinase enabling Fas-mediated apoptosis [113], while KHDRBS1 regulates exon 3 inclusion of the anti-apoptotic protein survivin that is higher expressed in advanced breast cancers [114]. The relation between splice factor levels and the ratio between the pro-apoptotic Bcl-Xs and anti-apoptotic Bcl-Xl splice variants is less evident. The activity of the splice factors PTBP1 [115], hnRNPF/H [116] and KHDRBS1 [117] increase the expression of Bcl-Xs, whereas hnRNPK favors Bcl-Xl expression [118]. In the end, the overall altered splicing in transformed cells is likely defined by different key factors which most probably changes during the different stages of cancer progression.

Transformation of normal cells into cancer cells almost invariably results in reduced genome stability. Tumor cells adapt to the load of mutations by activation of the DNA damage response (DDR) which prevents further proliferation and requires extra time to repair the lesions and might even result in apoptosis. Interestingly, splice factors TRA2α and TRA2β are clearly upregulated in breast cancer and those oncogene-like factors limit the amount of DNA damage thereby preventing cell death before entering the G2 phase. Indeed, dual knockdown of these factors results in a decreased expression of full length of CHEK1 (G2 checkpoint protein), leading to increased levels of the DNA damage marker γH2AX and decreased cell viability [77]. Moreover, upon DNA damage DDX54 interacts with pre-mRNAs containing introns with weak acceptor splice sites, leading to lower intron retention and increased survival [119].

#### Drug resistance

In addition to resisting cell death due to physiological stresses, cancer cells might also gain properties resulting in resistance to cytotoxic agents. For example, overexpression of RNA-binding protein and splice factor RBM17 occurs in many cancer types and is associated with drug resistance to general chemotherapeutic agents such as doxorubicin and vincristine [120]. Next, SRPK1 inhibition increased apoptotic potential and cell killing when combined with gemcitabine and cisplatin treatments through impaired MAPK1, MAPK3 and PI3K pathways [121]. Furthermore, TRA2A overexpression results in AS of RSRC2 and decreased protein expression, contributing to paclitaxel resistance in triple-negative breast cancer patients [122]. Low levels of PRPF4B correlate to patient acquired resistance to microtubule targeting chemotherapeutics, presumably by regulating the spindle assembly checkpoint [69]. Moreover, the subunits of the SF3B complex SF3B1 and SF3B3 are upregulated in ER-α positive cells with acquired tamoxifen and fulvestrant resistance, with SF3B3 overexpression relating to a decrease in overall survival [64]. Opposite to the previous factors, spliceosome component SRSF4 induces splicing events followed by apoptosis in cancer cells when combined with the cytotoxic agent cisplatin. Knockdown of this factor reverses these splicing events and as a result significantly reduces cisplatin induced apoptosis [123]. Interestingly, this confirms that there is a dual role for different splice factors in apoptosis regulation.

### Deregulating cellular energetics

Closely related to uncontrolled cell proliferation is the deregulation of cellular energetics, which is necessary to feed cells during growth and division. In aerobic conditions, healthy cells fuel their energy by processing glucose through glycolysis in the cytoplasm and oxidative phosphorylation in the mitochondria. Because the mitochondria consume high amounts of oxygen, energy production in anaerobic conditions relies only on glycolysis. However, cancer cells can reprogram their glucose metabolism using mainly glycolysis even in the presence of oxygen, named the Warburg effect. Splice factors that are suggested to control the Warburg effect are multiplayers PTBP1, hnRNPA1 and hnRNPA2 which not surprisingly are also involved in breast cancer growth and invasion. All three factors favor pyruvate kinase exon 10 inclusion causing higher levels of the M2 isoform (PKM2) compared to M1 (PKM1) resulting in decreased oxygen consumption contributing to the Warburg effect [76, 124]. PTBP1, hnRNPA1, hnRNPA2 levels are regulated by MYC [124]. Interestingly, hnRNPA1 also regulates MYC by regulating AS of the MYC-interacting protein Max, resulting in increased Delta Max levels in glioblastoma. Delta Max but not Max stimulates the expression of glycolytic genes and is required for tumor growth *in vivo* [125].

PTPBP1, hnRNPA1 and hnRNPA2 are currently the only splice factors that have been related to the Warburg effect. However, since hypoxia is driving AS in breast cancer cells [126] and other splicing events of key metabolic genes such as PFKFB4 – which is responsible for retaining fructose-2,6-biphosphate, a key regulator of glycolysis – are altered in tumor tissue [127], we hypothesize that more spliceosome components are involved in cancerous cell metabolism.

### Angiogenesis

New blood vessel formation or angiogenesis is critical for tumor progression since it i) provides the tumor with nutrients needed for growth and ii) brings the tumor cells in close proximity to blood circulation facilitating invasion and metastasis formation. Vascular endothelial growth factor (VEGF) is a key component in both physiological and pathological angiogenesis. Breast cancer patients with elevated VEGF levels have a higher risk to develop metastases or death compared to other patients [128] and therefore inhibition of this factor is a promising therapeutic strategy [129]. VEGF can be alternatively spliced by using a distal splice site selection in exon 8, resulting in the anti-angiogenic isoform VEGF_xxx_b bearing a different C-terminus [130–132]. Splice site selection is dependent on SRPK1/2 phosphorylation of the RNA-binding splice factor SRSF1. Furthermore, SRPK1 regulates VEGF splicing and activity in prostate cancer: SRPK1 knockdown results in up-regulation of the anti-angiogenic isoform VEGF_xxx_b and decreased angiogenesis in a xenograft model [133]. Accordingly, mutations in the tumor suppressor gene WT1 lead to increased SRPK1 levels and hyper phosphorylated SRSF1, reducing anti-angiogenic VEGF_xxx_b levels [134]. Treatment with SRPK1/2 inhibitors results in reduced angiogenesis, suggesting that AS regulation might provide a promising strategy to inhibit angiogenesis through depletion of pro-angiogenic components such as VEGF [135]. This is confirmed by the prediction that targeting of specific VEGF isoforms might be the best strategy to reduce free VEGF in tumors [136]. Another example is the splicing regulator Nova2 that is involved in vascular lumen formation, an essential step in angiogenesis. Nova2 targets exons implicated in the partitioning-defective (Par) complex and its regulators including Par3, Arhgef6 and Rapgap1. The Par complex interacts with tight junctions and cadherins and is important for lumen formation by endothelial cells, thereby being essential for cellular and tissue homeostasis [137–140]. Nova2 knockdown interferes with vascular lumen development *in vivo* and impairs endothelial cell polarity [137]. Although Nova2 has not been linked to tumorigenesis yet, it might be a potential target to inhibit angiogenesis.

### Splice factors in the other hallmarks

In the last decade, splice factors levels have extensively been related to five hallmarks (Figure 1), suggesting a strong relation between splicing regulation and cancer development and progression. However, the remaining five hallmarks (genomic instability, tumor promoting inflammation, enabling replicative immortality, avoiding immune destruction and evading growth suppression) are still unaddressed. Here, we used RNA sequencing data from primary breast tumors from The Cancer Genome Atlas to investigate the potential role of splice factors in these hallmarks. For all of these hallmarks, we selected representative genes based on literature (Figure 2A, Supplementary Table 4) and calculated their log2 fold change (FC) between normal and tumor tissue. Key representative genes were selected based on their differential expression between normal and tumor tissue. For example, genome instability is characterized by a loss of repair mechanisms [21]. Comparing normal and primary tumor expression levels for genes involved in repair mechanisms, we identified four genes involved in nucleotide excision repair to be significantly downregulated in tumor tissue (Figure 2B). Next, we selected ten patients that were not affected (control patients) and ten patients that were heavily affected (hallmark patients) by the hallmark of interest (Figure 2C for genome instability). Finally, splice factor expression levels were compared between control and hallmark patients using a student’s t-test and after correction for multiple testing, splice factors significantly related to the specific hallmark could be identified (Supplementary Table 3). Interestingly, we could detect splice factors related to all remaining hallmarks, except for tumor promoting inflammation. Some of these splicing factors have already been associated with other hallmarks. EFTUD2 expression levels are linked to markers of replicative immortality while it was previously shown to interact with ER and affect breast cancer proliferation [67]. Loss of WDR77 resulted in loss of proliferative genes and expression levels are linked to genome instability [79]. Interestingly, we also identified splice factors that have not been linked to other hallmarks in breast cancer before, such as CRNKL1, RALY and JUP. Future functional studies can use our analysis as a starting point to unravel the causal relationship between splice factor levels and these hallmarks of cancer.

## FUTURE PERSPECTIVES AND CONCLUSION

As splice factors are frequently overexpressed in cancer compared to normal tissue, but also in highly invasive compared to less invasive tumors, splice factors might be a new promising therapeutic avenue in preventing breast cancer metastasis thereby lowering mortality in women. A possible drawback of inhibiting these factors could be the generation of adverse side effects when considering their critical function in intron removal in normal tissue. However, recent studies demonstrate that splice factor inhibition might certainly be applicable to the clinical situation. Both tumor-bearing mice and pre-diagnostic human samples of ER positive and triple negative breast cancer demonstrate autoantibody reactivity against spliceosomal proteins suggesting that at least partial inhibition for some of these factors should be possible [141, 142]. Furthermore, screens with natural products with antitumor characteristics resulted in the identification of spliceosome targeting drugs exhibiting cytostatic effects in multiple tumor cell lines by causing cell cycle arrest in G1 and G2/M phase [143–145]. Antitumor activities were confirmed in animal models and remarkably, general cytotoxicity was not observed. Moreover, these potential drugs seem to be more effective in cancer cells with some of them even targeting multidrug-resistant cells [146–148]. Recently, the natural compound resveratrol demonstrated to inhibit the oncogenic splice factor hnRNPA1 by inducing tumor suppressive miRNAs miR-424 and miR-503 via p53 thereby controlling tumor growth [149]. Also treatment with the CLK inhibitor T-025 reduced SR protein phosphorylation, which resulted in general effects on exon skipping and reduced cancer cell growth *in vitro* and *in vivo* [150]. Of note, most of the drugs described in the literature target the SF3B complex, a five-polypeptide subcomplex of the spliceosomal U2 snRNP. Small molecules affecting spliceosomal function by inhibiting different splicing complexes are known [151], but their potential role in combatting (breast) cancer has to be investigated. Other interesting targets would be the SR proteins or its upstream kinases like SRPK1 that has been demonstrated to be critical in multiple steps of breast cancer progression and for which inhibitors have been developed [105, 133, 152, 153]. Finally, pharmacological inhibition of the spliceosome would be a promising therapeutic strategy for MYC-addicted breast cancer tumors.

Next to the use of small molecules, the potential of using splice-switching antisense oligonucleotides (SSOs) to modulate AS is of great therapeutic interest. SSOs are single-stranded oligonucleotides consisting of 20-30 nucleotides that bind to pre-mRNA and sterically prevent splicing factor binding, resulting in splice site switching. In contrast to normal antisense oligonucleotides (ASOs), SSOs are chemically modified to prevent breakdown of the targeted transcript to specifically target splicing without affecting total transcript levels [154]. Furthermore, SSOs are easy to synthesize and deliver, are relatively stable and can enter many different cell types [155, 156]. Although the use of SSOs was initially mainly studied in neuromuscular diseases such as Duchenne muscular dystrophy and spinal muscular atrophy [157], the therapeutic potential of SSOs as anti-cancer therapy is currently widely exploited [158]. For example, treatment with SSO stimulating exon 11 skipping caused better response to PARP inhibitors by inducing DNA double strand breaks [159]. Additionally, treatment with SSOs inducing exon 15 HER2 skipping caused downregulation of full length HER2 resulting in decreased downstream signaling, reduced cell proliferation and induction of apoptosis [160]. As a next step, clinical studies have to prove the use of these SSOs as anti-cancer therapy in breast cancer patients.

Since 94% of human genes are alternatively spliced [161, 162], splicing can be a very powerful new layer of oncogenic control. As discussed in detail above, splice factors demonstrated to play major roles in different hallmarks of cancer during tumorigenesis (Figure 1 and Supplementary Table 2) and some of these factors were already described as new oncogenes or tumor suppressors. Since the role of many splice factors is not limited to a specific step or cancer hallmark they might provide a new approach to combat (breast) cancer progression. However, there are still many AS events that have been associated with cancer progression which cannot be attributed to specific splice factors yet. The introduction of the RNAi libraries and more recently the CRISPR Cas9 technology together with the development of high-throughput screening technologies [105, 163–165] would allow systematic evaluation of spliceosomal components in multiple aspects of breast cancer progression, such as proliferation and migration. Future studies should apply these technologies to uncover the complete signaling landscape of splice factors in breast cancer progression that then can be used to develop specific splice factor inhibitors preventing metastasis formation and patient deaths.

## SUPPLEMENTARY MATERIALS







## Abbreviations

AR: androgen receptor
AS alternative splicing; DDR: DNA damage response
EGFR: epidermal growth factor receptor
EMT: epithelial-to-mesenchymal transition
ER: estrogen receptor
FC: fold change
HER2: human epidermal growth factor receptor 2
HMEC: human mammary epithelial cell
Par: partitioning-defective
PK: pyruvate kinase
RBM4: RNA-binding motif protein 4
snRNP: small nuclear ribonucleoprotein
SR: serine/arginine-rich
SRPK1: splicing factor SRSF protein kinase 1
SRSF1: serine and arginine splicing factor 1
VEGF: vascular endothelial growth factor
