# Supplementary material for: Splicing regulatory factors in breast cancer hallmarks and disease progression
Source: Oncotarget. 2019 Oct 15;10(57):6021–37. doi: 10.18632/oncotarget.27215 (PMC6800274; doi:10.18632/oncotarget.27215)
Supplement: Supplementary file 1 [file oncotarget-10-6021-s001.pdf]

Splicing regulatory factors in breast cancer hallmarks and disease progression

SUPPLEMENTARY MATERIALS

Supplementary Table 1: Splice factor mutations in primary breast tumors. % mutated tumors was extracted from the COSMIC database. Driver genes were determined by the IntOGen database, in which mutations in driver genes were enriched during tumor development. See Supplementary Table 1

Supplementary Table 2: Splice factors in hallmarks of breast cancer. Overview splice factors affecting the different hallmarks in breast cancer. See Supplementary Table 2

Supplementary Table 3: Splice factors related to other hallmarks in breast cancer

Hallmark	Related splicing factors (Padj < 0.05)
Tumor-promoting inflammation	-
Enabling replicative immortality	SMNDC1, USP39, EFTUD2, PPIL1, ELAVL1
Genome instability	CCAR2, RBM5, WDR77
Avoiding immune destruction	QKI, CRNKL1, HNRNPF, LSM6, JUP, YBX1
Evading growth suppressors	ALYREF, DDX41, ILF2, PPIL2, RALY, SNRNPC

Supplementary Table 4: General markers for different cancer hallmarks

Hallmark	Marker	Subprocess	Selected
Tumor-promoting inflammation	CD168	Tumor associated macrophages	Yes
Tumor-promoting inflammation	CD63	Tumor associated macrophages	Yes
Enabling replicative immortality	TERT	Telomere maintenance and regulation	Yes
Enabling replicative immortality	TRF1	Telomere maintenance and regulation	Yes
Enabling replicative immortality	TRF2	Telomere maintenance and regulation	Yes
Enabling replicative immortality	POT1	Telomere maintenance and regulation	Yes
Enabling replicative immortality	TIN2	Telomere maintenance and regulation	Yes
Enabling replicative immortality	RAP1	Telomere maintenance and regulation	Yes
Enabling replicative immortality	TPP1	Telomere maintenance and regulation	Yes
Genome instability	DDB2	Nucleotide excision repair	Yes
Genome instability	ERCC1	Nucleotide excision repair	No
Genome instability	ERCC2	Nucleotide excision repair	No
Genome instability	ERCC3	Nucleotide excision repair	No
Genome instability	ERCC4	Nucleotide excision repair	No
Genome instability	ERCC5	Nucleotide excision repair	Yes
Genome instability	XPA	Nucleotide excision repair	Yes
Genome instability	XPC	Nucleotide excision repair	Yes
Genome instability	BRCA1	Double-strand break repair	No
Genome instability	TP53BP1	Double-strand break repair	No
Genome instability	TRIM28	Double-strand break repair	No
Genome instability	XRCC4	Double-strand break repair	No
Genome instability	APEX1	Base excision repair	No
Genome instability	APEX2	Base excision repair	No
Genome instability	FEN1	Base excision repair	No
Genome instability	PNKP	Base excision repair	No
Genome instability	MSH2	DNA mismatch repair	No
Genome instability	MSH3	DNA mismatch repair	No
Genome instability	MSH6	DNA mismatch repair	No
Genome instability	PMS2	DNA mismatch repair	No
Avoiding immune destruction	IL2RA	CTL activation	Yes
Avoiding immune destruction	CD38	CTL activation	Yes
Avoiding immune destruction	CD69	CTL activation	Yes
Avoiding immune destruction	HLA-DRA	CTL activation	Yes
Avoiding immune destruction	IFNG	CD4+ Th1 cells	Yes
Avoiding immune destruction	TNF	CD4+ Th1 cells	Yes
Avoiding immune destruction	IL-2	CD4+ Th1 cells	Yes
Evading growth suppressors	RB1	Tumor suppressors	Yes
Evading growth suppressors	TP53	Tumor suppressors	No
Evading growth suppressors	APC	Tumor suppressors	No
Evading growth suppressors	BRCA1	Tumor suppressors	No
Evading growth suppressors	BRCA2	Tumor suppressors	No
Evading growth suppressors	PTEN	Tumor suppressors	Yes
Evading growth suppressors	WT1	Tumor suppressors	Yes
Evading growth suppressors	WT2	Tumor suppressors	No
Evading growth suppressors	NF1	Tumor suppressors	Yes
Evading growth suppressors	NF2	Tumor suppressors	No
